# Too close for comfort? COVID-19-related stress among older couples and the moderating role of closeness

**DOI:** 10.1093/geroni/igab046.3744

**Published:** 2021-12-17

**Authors:** Kira Birditt, Angela Turkelson, Angela Oya

**Affiliations:** University of Michigan, Ann Arbor, Michigan, United States

## Abstract

Married and cohabiting couples have important influences on one another’s stress and well-being. Pandemic-related stress may influence the extent to which couples' stress levels are coregulated. This study examined the experience of nonspecific stress and pandemic-related stress and the moderating role of closeness among couples aged 50 and over in which at least one member had hypertension. A total of 30 couples reported their feelings of closeness to one another in a baseline interview and their feelings of nonspecific stress and pandemic-related stress every three hours for 5 days. There was no difference in closeness and nonspecific stress between husbands and wives. Wives reported greater pandemic-related stress than husbands. Actor-partner interdependence models revealed that wives’ nonspecific stress predicted husbands’ nonspecific stress (b = 0.17, SE = 0.04, p < .001) and that husbands’ nonspecific stress predicted wives’ nonspecific stress in each three hour period (b = 0.19, SE = 0.04, p < .001) and these associations were not moderated by closeness. Coregulation in pandemic-related stress among husbands and wives was moderated by wives’ feelings of closeness such that when wives’ feelings of closeness were lower, greater husband pandemic-related stress predicted lower pandemic-related stress for wives (b = -0.16, SE = 0.07, p < .05) whereas when wives’ feelings of closeness were higher, greater husband pandemic-related stress predicted greater pandemic-related stress for wives (b = 0.22, SE = 0.09, p < .05). These findings indicate that closeness may have detrimental effects especially when considering emotional coregulation in couples regarding the pandemic.

